# Quantitative Lung Ultrasonography to Guide Surfactant Therapy in Neonates Born Late Preterm and Later

**DOI:** 10.1001/jamanetworkopen.2024.13446

**Published:** 2024-05-28

**Authors:** Daniele De Luca, Luca Bonadies, Almudena Alonso-Ojembarrena, Diletta Martino, Irene Gutierrez-Rosa, Barbara Loi, Reedhi Dasani, Letizia Capasso, Eugenio Baraldi, Alexis Davis, Francesco Raimondi

**Affiliations:** 1 Division of Pediatrics and Neonatal Critical Care, “A. Béclère” Hospital, AP-HP–Paris Saclay University, Paris, France; 2 Physiopathology and Therapeutic Innovation Unit–INSERM U999, Paris Saclay University, Paris, France; 3 Neonatal Intensive Care Unit, Department of Women’s and Children’s Health, University Hospital of Padova and Institute of Pediatric Research “Città della Speranza,” Padua, Italy; 4 Neonatal Intensive Care Unit, Puerta del Mar University Hospital, Cádiz, Spain; 5 Biomedical Research and Innovation Institute of Cádiz (INiBICA), Research Unit, Puerta del Mar University Hospital, Cádiz, Spain; 6 Department of Pediatrics, Division of Neonatal and Developmental Medicine, Stanford University, School of Medicine, Palo Alto, California; 7 Division of Neonatology, Department of Translational Medical Sciences, University of Naples Federico II, Naples, Italy

## Abstract

**Importance:**

Surfactant administration may be needed in late preterm through full-term neonates, but the pathophysiology of their respiratory failure can be different from that of early preterm neonates. The lung ultrasonography score (LUS) is accurate to guide surfactant replacement in early preterm neonates, but to our knowledge, it has not yet been studied in the late preterm through full-term neonatal population.

**Objective:**

To assess whether LUS is equally accurate to predict surfactant need in late preterm through full-term neonates as in early preterm neonates.

**Design, Setting, and Participants:**

This prospective, international, multicenter diagnostic study was performed between December 2022 and November 2023 in tertiary academic neonatal intensive care units in France, Italy, Spain, and the US. Late preterm through full-term neonates (≥34 weeks’ gestation) with respiratory failure early after birth were enrolled.

**Exposure:**

Point-of-care lung ultrasonography to calculate the neonatal LUS (range, 0-18, with higher scores indicating worse aeration), which was registered in dedicated research databases and unavailable for clinical decision-making.

**Main Outcomes and Measures:**

The main outcomes were the area under the curve (AUC) in receiver operating characteristic analysis and derived accuracy variables, considering LUS as a replacement for other tests (ie, highest global accuracy) and as a triage test (ie, highest sensitivity). Sample size was calculated to assess noninferiority of LUS to predict surfactant need in the study population compared with neonates born more prematurely. Correlations of LUS with the ratio of hemoglobin oxygen saturation as measured by pulse oximetry (SpO_2_) to fraction of inspired oxygen (FiO_2_) and with the oxygen saturation index (OSI) were assessed.

**Results:**

A total of 157 neonates (96 [61.1%] male) were enrolled and underwent lung ultrasonography at a median of 3 hours (IQR, 2-7 hours) of life; 32 (20.4%) needed surfactant administration (pretest probability, 20%). The AUC was 0.87 (95% CI, 0.81-0.92). The highest global accuracy and sensitivity were reached for LUS values higher than 8 or 4 or lower, respectively. Subgroup analysis gave similar diagnostic accuracy in neonates born late preterm (AUC, 0.89; 95% CI, 0.81-0.97; n = 111) and early term and later (AUC, 0.84; 95% CI, 0.73-0.96; n = 46). After adjusting for gestational age, LUS was significantly correlated with SpO_2_:FiO_2_ (adjusted β, −10.4; 95% CI, −14.0 to −6.7; *P* < .001) and OSI (adjusted β, 0.2; 95% CI, 0.1-0.3; *P* < .001).

**Conclusions and Relevance:**

In this diagnostic study of late preterm through full-term neonates with respiratory failure early after birth, LUS accuracy to predict surfactant need was not inferior to that observed in earlier preterm neonates. An LUS higher than 8 was associated with highest global accuracy (replacement test), suggesting that it can be used to guide surfactant administration. An LUS value of 4 or lower was associated with the highest sensitivity (triage test), suggesting it is unlikely for this population to need surfactant.

## Key Points

**Question:**

Is the neonatal lung ultrasonography score (LUS) equally accurate to predict surfactant need in late preterm through full-term neonates as in neonates born more prematurely?

**Findings:**

In this multicenter diagnostic study of 157 late preterm through full-term neonates with respiratory failure early after birth, the accuracy of LUS in these neonates was similar to that observed in early preterm neonates. An LUS higher than 8 and 4 or lower had the highest global accuracy (replacement test) and highest sensitivity (triage test), respectively.

**Meaning:**

The findings suggest that the LUS can accurately guide surfactant administration in late preterm through full-term neonates with respiratory failure occurring shortly after birth.

## Teaser

This diagnostic study examines whether the neonatal lung ultrasonography score has equivalent accuracy to predict surfactant need in neonates born late preterm and later as in neonates born more prematurely.

## Introduction

Point-of-care lung ultrasonography is becoming widely used for its ease, noninvasiveness, and accuracy, as it allows a refined diagnosis of the main neonatal respiratory disorders.^1^ Ultrasonography findings may be assessed quantitatively using dedicated scores that can guide respiratory interventions,^2^ and a specific lung ultrasonography score (LUS) has been validated for neonatal use.^3^

Surfactant is a cornerstone of neonatal critical care and is the licensed treatment for respiratory distress syndrome (RDS).^4,5^ Since the early trials, surfactant administration has been based mainly on fraction of inspired oxygen (FiO_2_) levels.^6^ This policy spread thereafter as other tools to guide surfactant therapy appeared to be either cumbersome or inaccurate and to lack sufficient clinical development.^7^ Using FiO_2_ thresholds is, however, an oversimplified method since inspired oxygen is only one of the many factors influencing oxygenation.^7^ Moreover, oxygenation impairment is the last consequence in the pathophysiology cascade of events^8^; thus, FiO_2_ can increase after the optimal time window for surfactant administration (ie, the first 2-3 hours of life).^9^ Despite these drawbacks, FiO_2_ thresholds are still widely used to decide whether to administer surfactant.^7^

Ultrasonography-guided surfactant administration can allow personalized therapy and reduce delayed treatments.^10,11^ The LUS has been demonstrated to predict surfactant need in preterm and extremely preterm neonates with RDS treated with continuous positive airway pressure (CPAP),^3,12,13^ and meta-analyses reported the highest global accuracy with cutoff values between 6 and 8.^7,14,15^ Diagnostic accuracy might be different in neonates born more prematurely since RDS prevalence is inversely proportional to gestational age and, in these patients, respiratory failure may have different pathophysiology and be caused by other disorders, including transient tachypnea of the neonate (TTN). Nonetheless, some patients with TTN may also develop concomitant RDS due to relative surfactant deficiency and may benefit from surfactant replacement.^16^ In addition, late preterm through full-term neonates represent challenging cases as they are often delivered in level I or II perinatal centers and, in some areas, the occurrence of respiratory failure may demand their transfer to referral neonatal intensive care units (NICUs) for evaluation and surfactant treatment if needed. Though LUS may have the potential to guide surfactant replacement in late preterm through full-term neonates, to our knowledge, it has not yet been systematically studied in this population. We aimed to assess whether LUS is equally accurate to predict surfactant need in late preterm through full-term neonates as it is in neonates born more prematurely.

## Methods

### Study Design

This was an international, multicenter diagnostic accuracy study conducted in 5 referral NICUs in France, Italy, Spain, and the US between December 2022 and November 2023. The study was pragmatic as it used only data routinely obtained during clinical care that were not changed for study purposes. Ethical approval was granted in each participating center, and if required by local regulations, written or oral parental informed consent was obtained at NICU admission. Data were prospectively collected in a dedicated, secured, and deidentified database for each participating hospital and subsequently merged at Paris Saclay University, which served as the coordinating center. Relevant privacy regulations were respected. Manuscript preparation followed the Standards for Reporting of Diagnostic Accuracy (STARD) guideline.^17^

### Participants

Neonates admitted to the NICU within the first 72 hours of life for respiratory failure were consecutively enrolled if their gestational age was 34 weeks or more. Gestational age was considered based on the best obstetric estimate. Respiratory support consisted of nasal mask– or binasal prongs–delivered CPAP set at 5 to 6 cm H_2_O as per local practice and was started when patients had dyspnea (ie, Silverman score ≥1) with need for supplemental oxygen to achieve hemoglobin oxygen saturation as measured by pulse oximetry (SpO_2_) of 90% or greater. Conversely, when ongoing resuscitation was needed, invasive ventilation was used per local practice. Supplemental oxygen was added when the respiratory support in room air was insufficient to achieve SpO_2_ of 90% or greater. The remaining perinatal management was essentially based on current international guidelines.^18,19^ Surfactant (poractant alfa, 200 mg/kg) was administered when FiO_2_ was persistently greater than 0.30, as currently advised.^20^ Ultrasonography findings were only considered qualitatively for diagnostic or educational purposes,^1^ and LUS was not used to decide surfactant administration or any clinical intervention. The type of respiratory failure was diagnosed according to prespecified integrated consensus criteria based on perinatal history, biology, and clinical evolution according to the Montreux consensus criteria (eTable 1 in Supplement 1)^21^ and was classified as RDS, TTN, or neonatal acute respiratory distress syndrome (NARDS). Exclusion criteria were major congenital malformations or chromosomal anomalies, air leaks (ie, pneumothorax, pneumomediastinum) preventing comprehensive ultrasonography visualization of the lung parenchyma, surgery during the first week of life, hemodynamic instability (defined as need for any inotrope), congenital surfactant anomalies, pulmonary hypoplasia or congenital lung malformation, persistent pulmonary hypertension (defined as need for nitric oxide or other pulmonary vasodilators), and need for extracorporeal life support.

### Index Test and Reference Standard

The LUS was the index test, calculated at NICU admission and always before surfactant administration, if administered. Ultrasonography was performed with microlinear, hockey stick–shaped, high-frequency (15-18 MHz) probes; the machine setting was as previously described.^22^ Lung ultrasonography scores were calculated on 6 thoracic areas (3 per each hemithorax [upper and lower anterior and lateral]), assigning to each area a value of 0 to 3 based on classic ultrasonography semiology (0 for normal, 1 for interstitial-alveolar, 2 for severe interstitial-alveolar [ie, white lung] pattern, and 3 for consolidated areas), as originally published.^3^ Thus, the score ranges from 0 (best aeration) to 18 (worst aeration). The score was calculated by investigators proficient in the technique (ie, with at least 1 year of lung ultrasonography experience) (L.B., A.A.-O., D.M., I.G.-R., B.L., and L.C.). The LUS was registered in dedicated research databases, which were unavailable for clinical decision-making and were not used to indicate surfactant administration. This strategy was previously applied^3^ and considered the best way to mask LUS since perfect blinding was impossible as lung ultrasonography is routinely used in the participating centers. In detail, clinicians not performing ultrasonography were unaware of the LUS, but it was impossible to conceal patient conditions, such as vital monitoring and clinical appearance, to investigators performing ultrasonography. Nonetheless, previous studies have demonstrated equally optimal interobserver agreement for lung ultrasonography interpretation with or without operators’ blinding.^3,23,24^ Lung ultrasonography values of 6 and 8 were the prespecified positive cutoffs, as these have been associated with the highest global accuracy in early preterm neonates^7,14^; thus, we used them to investigate noninferiority in the study population. The reference standard was an FiO_2_ level of 0.30, as this is the threshold suggesting surfactant administration in the European guidelines^20^ and is widely used.

Preductal SpO_2_ was measured with artifact-filtering monitors when the signal was regularly smooth and was registered together with FiO_2_ in the patient’s electronic file per local NICU policies. Oxygenation was described using the SpO_2_:FiO_2_ ratio and the oxygen saturation index (OSI; calculated as mean airway pressure × FiO_2_:SpO_2_) assessed at the time of lung ultrasonography. For nonintubated neonates, the CPAP level was considered as the mean airway pressure and leaks were reduced with patient positioning and gentle mouth closure.

### Statistical Analysis

The statistical plan was decided before the end of the study and is available in the IRSCTN registry.^25^ For an LUS in late preterm through full-term neonates to be as accurate as it is in early preterm neonates, receiver operating characteristic (ROC) analysis should give a similar area under the curve (AUC). We set a target AUC of 0.93 (95% CI, 0.86-0.99), as this was originally found to guide surfactant administration with LUS in preterm neonates.^3^ We considered an AUC of 0.80 as the null hypothesis (ie, we considered global accuracy in the study population to be inferior to that in neonates born more prematurely if the AUC was <0.80) since this is the value reported to guide surfactant replacement with FiO_2_ in preterm neonates.^7^ The proportion of late preterm through full-term neonates with respiratory failure needing surfactant treatment was considered to be 20%, as previously reported.^3^ Power was set at 80% and α at 0.05. With these parameters, the needed sample size was a cohort of 145 patients (29 positive cases and 116 negative cases).^26^

Clinical characteristics were compared between study participants who did and did not receive surfactant using χ^2^, Fisher exact, *t*, or Mann-Whitney *U* tests, as appropriate. The ROC analysis was performed, and derived diagnostic accuracy parameters (sensitivity, specificity, positive and negative likelihood ratios, positive and negative predictive values, global accuracy, and positive and negative posttest probability) were calculated with their 95% CIs.

The AUC (and derived diagnostic parameters) was our main outcome, and we evaluated LUS as a replacement for other tests—that is, with the highest sensitivity and specificity (ie, highest global accuracy) possible. Additionally, we investigated the reliability of LUS as a triage test—that is, with the highest sensitivity irrespective of specificity.^27^ The ROC analysis was performed for the whole population and for 2 prespecified subgroups represented by patients born late preterm and early term and later (ie, with gestational age between 34 and 36 6/7 weeks or 37 weeks or more, respectively) to investigate the effect of gestational age on diagnostic accuracy.^27^ The AUC was compared between subgroups, with AUCs originally reported in early preterm neonates^3,12^ and with the summary AUC obtained by a recent meta-analysis^15^ using the Hanley method.^26^

Finally, the correlation between LUS and oxygenation metrics was investigated with Spearman correlation coefficients and adjusted for gestational age using linear regression.^27^ Multicollinearity was evaluated as previously published.^28^ Analyses were performed with SPSS, version 29 (IBM Corp) and MedCalc, version 13.3 (MedCalc Software Ltd), and 2-sided *P* < .05 was considered significant.

## Results

**Figure 1.**
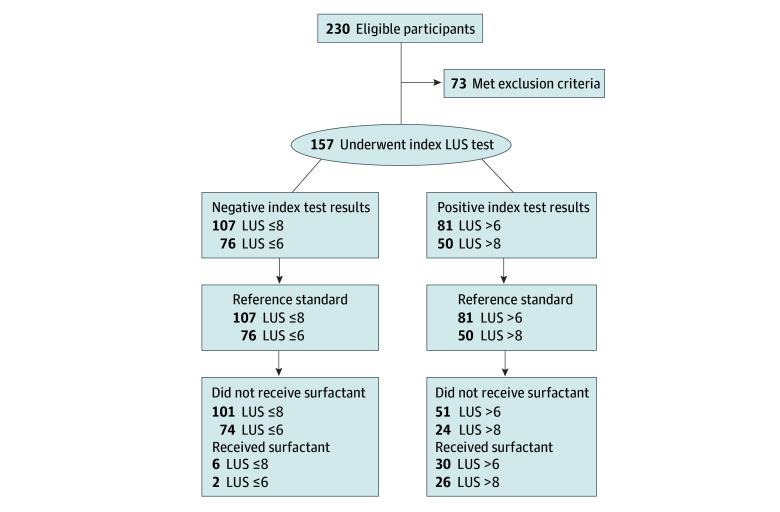
STARD Diagram There were no indeterminate results and no missing data for the index test and the reference standard (ie, all enrolled neonates underwent lung ultrasonography score [LUS] calculation and fraction of inspired oxygen [FiO_2_] monitoring; surfactant was always given when FiO_2_ was persistently >0.30). Two prespecified LUS positivity cutoff values (>6 or >8) were used for the index test when considering it as a replacement test, as these values are associated with the highest global diagnostic accuracy in neonates born at less than 34 weeks’ gestation.^7,14^ Additionally, the analysis allowed identification of which LUS cutoff values were associated with the highest sensitivity to use LUS as a triage test.

Figure 1 shows the study flowchart; the index test (LUS) and reference standard (FiO_2_) had no missing or indeterminate data, and the same applied to surfactant data. All patients completed the study. Table 1 and eTable 2 in Supplement 1 give basic population details; 157 neonates were enrolled (mean [SD] gestational age, 35.7 [2.3] weeks; 61 [38.9%] female, 96 [61.1%] male). Patients who needed surfactant had worse oxygenation metrics and LUS compared with those who did not. Lung ultrasonography was conducted at a median of 3 hours (IQR, 2-7 hours) of life. Thirty-two neonates (20.4%) needed surfactant administration (pretest probability, 20%). At the time of ultrasonography, 145 neonates (92.4%) were supported by CPAP and 12 (7.6%) received invasive ventilation. Forty-eight (30.6%), 93 (59.2%), and 16 (10.2%) neonates were diagnosed with RDS, TTN, and NARDS, respectively; 24 (50.0%) with RDS, 2 (2.2%) with TTN, and 6 (37.5%) with NARDS received surfactant. Only 2 neonates (1.3%) developed signs of respiratory failure beyond the first day of life (one at 40 hours and another at 72 hours of life). NARDS was triggered by meconium aspiration and perinatal infection in 12 cases (75.0%) and 4 cases (25.0%), respectively. Surfactant administration occurred at a median postnatal age of 6 hours (IQR, 3-10 hours) of life. All but 2 neonates (1.3%) survived; median NICU stay was 6 days (IQR, 3-12 days).

**Table 1.** Demographics and Clinical Details

Characteristic	Neonates^a^			*P* value^b^
	Whole cohort (N = 157)	Surfactant given (n = 32)	No surfactant (n = 125)	
Gestational age, mean (SD), wk	35.7 (2.3)	35.6 (2.3)	35.7 (2.3)	.85
Birth weight, mean (SD), g	2557 (748)	2524 (672)	2567 (768)	.76
Sex				
Female	61 (38.9)	9 (28.1%)	52 (41.6)	.23
Male	96 (61.1)	23 (71.9)	73 (58.4)	
SGA	19 (12.1)	7 (21.9)	12 (9.6)	.11
Prenatal steroids^c^	60 (38.2)	10 (31.3)	41 (32.8)	.61
Cesarean delivery	96 (61.1)	19 (59.4)	77 (61.6)	.98
5-min Apgar score, median (IQR)^d^	8 (7-9)	8 (7-9)	8 (7 9)	.42
Measure at enrollment, median (IQR)				
Postnatal age, h	3 (2-7)	3 (2-6)	4 (2-7)	.68
FiO_2_^d^	0.21 (0.21-0.30)	0.32 (0.25-0.44)	0.21 (0.21-0.25)	<.001
Airway pressure, mean (SD), cm H_2_O	6.8 (3.4)	7.8 (2.7)	6.6 (3.5)	.07
OI^d^^,^^e^	2.3 (1.7-3.3)	4.5 (1.9-5.2)	2.1 (1.6-3.1)	.03
SpO_2_:FiO_2_^d^	404 (308-457)	281 (198-366)	438 (368-457)	<.001
OSI^d^^,^^f^	1.6 (1.3-2.2)	2.2 (1.7-3.3)	1.4 (1.3-1.7)	<.001
Paco_2_^g^	46 (39-54)	48 (42-68)	46 (39-53)	.33
LUS^d^	7 (3-9)	12 (9-12)	6 (3-8)	<.001

Abbreviations: FiO_2_, fraction of inspired oxygen; LUS, lung ultrasonography score; OI, oxygenation index; OSI, oxygen saturation index; Paco_2_, partial pressure of carbon dioxide; SGA, small for gestational age; SpO_2_, oxygen saturation as measured by pulse oximetry.

^a^ Data are expressed as number (percentage) of neonates unless otherwise indicated. Patients were enrolled and underwent lung ultrasonography at neonatal intensive care unit admission.

^b^ A *t* test or Mann-Whitney *U* test was used as appropriate.

^c^ Considered as two 12-mg betamethasone doses given at least 24 hours before birth.

^d^ Dimensionless variable.

^e^ Calculated as mean airway pressure  × FiO_2_:Pao_2_.

^f^ Calculated as mean airway pressure × FiO_2_:SpO_2_.

^g^ Values were measured according to Montreux consensus using arterial values or, if those were unavailable, transcutaneous or arterialized capillary values.^21^

**Figure 2.**
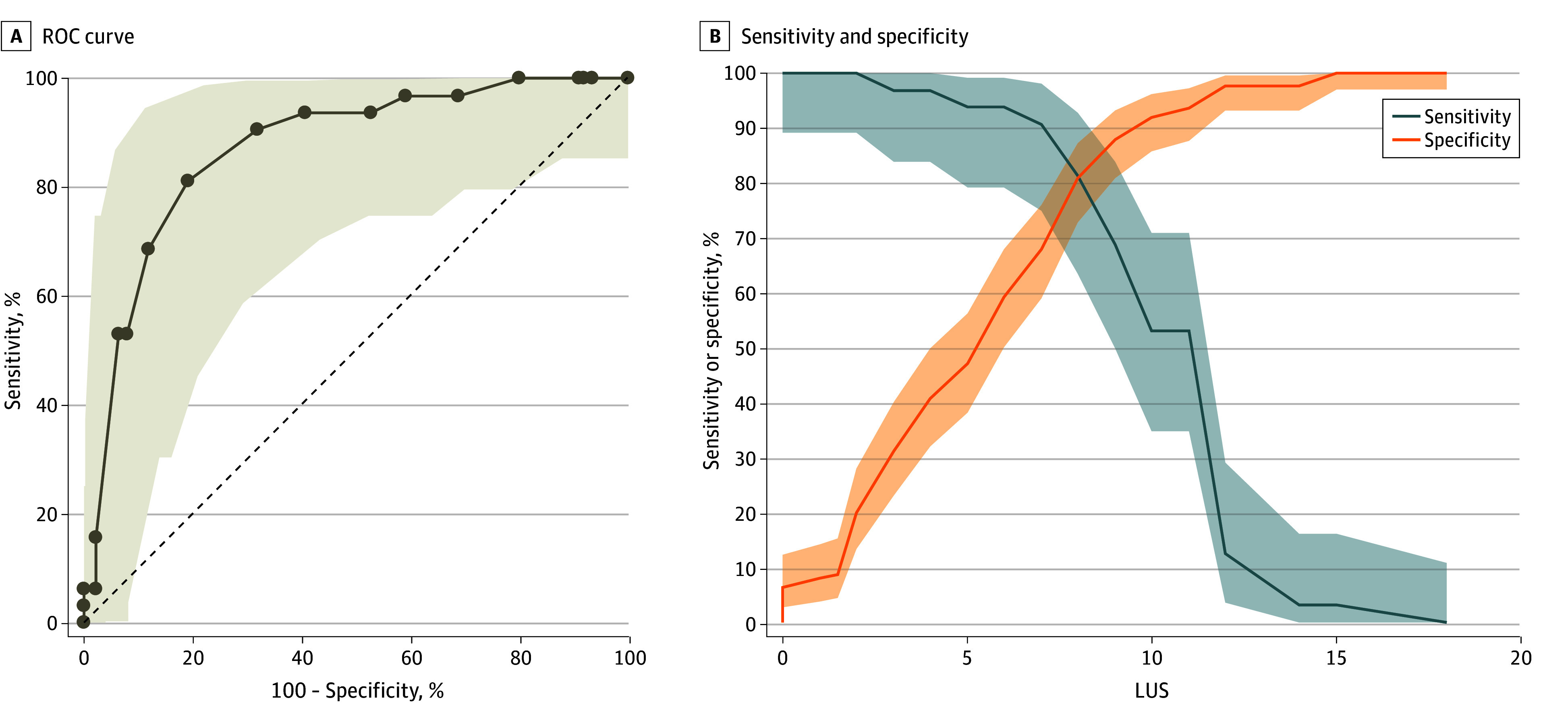
Receiver Operating Characteristic (ROC) Analysis for the Whole Population A, The ROC curve and 95% CI (shading) corresponded to an area under the curve of 0.87 (95% CI, 0.81-0.92). Diagonal line represents the chance line, corresponding to an area under the curve of 0.5. B, The crossing point is at an LUS score of 8. Shading indicates 95% CIs.

Figure 2 shows the ROC curve as well as sensitivity and specificity values for the entire study cohort. The AUC was 0.87 (95% CI, 0.81-0.92; *P* < .001), and the LUS cutoff associated with both the highest sensitivity and specificity—that is, the highest global accuracy (ie, LUS as a replacement test)—was 8 (Youden index, 0.65). The highest absolute sensitivity values (ie, LUS as a triage test) were reached for an LUS between 0 and 4 (sensitivity, 97%-100%). Table 2 and eTable 3 in Supplement 1 report the diagnostic accuracy parameters for the 2 prespecified cutoff values investigated for using LUS as a replacement test and as a triage test, respectively. A ROC analysis performed excluding patients with NARDS (AUC, 0.89; 95% CI, 0.82-0.96; *P* < .001) or those receiving invasive ventilation (AUC, 0.90; 95% CI, 0.84-0.96; *P* < .001) gave similar results.

**Table 2.** Diagnostic Accuracy Parameters for the 2 Prespecified LUS Thresholds Investigated as a Replacement Test

LUS threshold^a^	Sensitivity, % (95% CI)	Specificity, % (95% CI)	PLR (95% CI)	NLR (95% CI)	PPV, % (95% CI)	NPV, % (95% CI)	Global accuracy, % (95% CI)	Positive posttest probability, % (95% CI)	Negative posttest probability, % (95% CI)
>6	94 (79-99)	59 (50-68)	2.3 (1.8-2.9)	0.1 (0.03-0.4)	37 (32-43)	97 (91-99)	67 (64-75)	37 (31-42)	3 (8-9)
>8	81 (64-93)	81 (73-87)	4.3 (2.9-6.3)	0.2 (0.1-0.5)	52 (42-62)	94 (89-97)	82 (79-84)	52 (42-61)	5 (2-11)

Abbreviations: LUS, lung ultrasonography score; NLR, negative likelihood ratio; NPV, negative predictive value; PLR, positive likelihood ratio; PPV, positive predictive value.

^a^ These cutoff values were chosen to consider LUS as a replacement test since they are known to be associated with the highest global diagnostic accuracy (ie, the highest sensitivity and specificity) in neonates born at less than 34 weeks’ gestation.^7,14^

The AUC was not different from that originally described for preterm^3^ (AUC, 0.93; 95% CI, 0.86-0.99; comparison test *P* = .20) and extremely preterm^12^ (AUC, 0.94; 95% CI, 0.90-0.98; comparison test *P* = .07) neonates or from the summary AUC reported by a recent meta-analysis^15^ (AUC, 0.88; 95% CI, 0.82-0.91; comparison test *P* = .74). Subgroup analysis showed a similar diagnostic accuracy in neonates born late preterm (AUC, 0.89; 95% CI, 0.81-0.97; *P* < .001; n = 111) and early term and later (AUC, 0.84; 95% CI, 0.73-0.96; *P* < .001; n = 46); AUCs were similar between these 2 subgroups (*P* = .45). The LUS was significantly correlated with SpO_2_:FiO_2_ (ρ, −0.47; *P* < .001) and OSI (ρ, 0.42; *P* < .001) and remained so after adjustment for gestational age (SpO_2_:FiO_2_: adjusted β, −10.4; 95% CI, −14.0 to −6.7; *P* < .001; OSI: adjusted β, 0.2; 95% CI, 0.1-0.3; *P* < .001).

## Discussion

In this study, we found that the diagnostic accuracy of quantitative lung ultrasonography as a replacement test to predict surfactant need in late preterm through full-term neonates was comparable to that shown in patients born more prematurely. We also found that the technique was reliable as a triage test. These results were obtained using the same score and positive cutoff values^3,7,14^ and by comparing results with those previously reported in early preterm neonates.^3,7,12,14,15^ Our findings also found 8 to be the LUS cutoff associated with the highest global accuracy (replacement test) and indicated that values of 4 or lower had the highest sensitivity (triage test).

These and other characteristics make our findings coherent. The study was performed with a multicenter design including several centers with similarly established ultrasonography proficiency as well as comparable devices and practice. We also applied the best possible methods for a technique that was already embedded in routine clinical care within the point-of-care policy.^29^ The subgroup analysis found that gestational age did not significantly influence LUS diagnostic accuracy since similar AUCs were found in late preterm through full-term neonates. Consistently, there was an association between LUS-assessed lung aeration and oxygenation irrespective of patient age, and this is consistent with what our group previously reported in neonates born more prematurely.^3,12,30^ Additionally, ROC analysis was repeated after excluding patients with NARDS, for whom surfactant therapy is off label, and results were unchanged.

To our knowledge, this was the first study specifically dedicated to ultrasonography-guided surfactant administration in late preterm through full-term neonates, and the results are clinically relevant. Surfactant treatment in this population represents an open clinical problem since patients may be affected by different types of respiratory failure with variable surfactant deficiency or dysfunction, and these are difficult to assess at the bedside.^8^ Clinical, biologic, or imaging tests available to date have been either cumbersome or inaccurate, leaving surfactant treatment unguided.^8^ Quantitative lung ultrasonography is known to describe the lung volume available for gas exchange (ie, lung aeration) and has been validated against a number of techniques.^2,31^ In particular, lung aeration is correlated with surfactant adsorption early after birth,^32,33^ and this makes LUS pathobiologically sound to detect surfactant deficiency or dysfunction.

No clear literature guidance is available to date to guide surfactant administration in late preterm through full-term neonates with signs of respiratory failure. Our data indicated that if these patients had an LUS higher than 8, their probability to need surfactant was approximately 2 times higher (Table 2). This finding should inform clinical practice that has been largely based on FiO_2_ and clinical monitoring to date; an LUS higher than 8 in a neonate with respiratory failure in the first hours of life may be used with good accuracy to indicate surfactant administration (replacement test) and reduce delayed administration or at least personalize the clinical monitoring. Conversely, using quantitative lung ultrasonography with a lower LUS cutoff—that is, with higher sensitivity—may be useful to rule out surfactant need (triage test); a patient with an LUS of 4 or lower is unlikely to have worsening respiratory failure needing surfactant (eTable 2 in Supplement 1). Since LUS calculation is easy and not reliant on the operator’s expertise, its use as a triage test may be particularly important for neonates born in hospitals lacking advanced neonatal care and needing transfer to referral centers.^22^ Moreover, LUS calculation is not affected by patient transportation and can also be realized in mobile NICUs.^34^ Thus, LUS can help clinicians to reduce subjectivity when making decisions, such as neonatal transportation, that are associated with relevant consequences from the medical and public health perspective. This may be helpful to reserve NICU beds for patients who actually need them and is particularly important during disease outbreaks or resources shortage.^35^

### Limitations

This study has limitations. A multicenter design was needed to recruit enough patients since severe respiratory failure is relatively less common in more-mature neonates than in preterm neonates. The shared ultrasonography expertise and technique was also an asset, and since lung ultrasonography is relatively easy to learn,^36^ this may facilitate the applicability of our findings. The main limitation was that we used an FiO_2_ threshold as the reference standard to identify surfactant need, and this cannot be considered a gold standard for the aforementioned reasons. However, there is no consensus on a gold standard to identify surfactant deficiency or dysfunction at the bedside, and the FiO_2_ is the most widely used criterion; thus, our results are pragmatically useful.^8^ The studied sample size may seem relatively small but was comparable to that of previous studies recruiting preterm neonates^3,12^ and respected the targeted sample size calculation. We included neonates who already had signs of respiratory failure (ie, high suspicion index); thus, our data cannot support LUS to be used as a pure screening test in asymptomatic neonates. Previous studies on preterm populations have followed the same design, and surfactant replacement, when realized, occurred few hours after ultrasonography.^3,12^ This highlights the need for and difficulty of blinding the procedure and having a test quick enough to make clinical decisions in rapidly evolving situations. We acknowledge that our blinding was not perfect, but it was the best we could have provided for the nature of the studied intervention (ie, point-of-care ultrasonography), which unavoidably required observation of the patient. We do not know yet, however, whether LUS accuracy may be improved by repeating the examination—that is, if changes in LUS might be more accurate than a single assessment. Similarly, machine learning and artificial intelligence–assisted interpretation might improve the diagnostic accuracy and make LUS a type of automatized monitoring. Finally, we cannot clarify the effect of more modern respiratory support techniques on LUS, as these are usually reserved for preterm neonates^37,38^ and were not used in this study population. These issues warrant dedicated clinical studies to be elucidated.

## Conclusions

In this study, the diagnostic accuracy of LUS to predict surfactant need in late preterm through full-term neonates with respiratory failure shortly after birth was similar to that observed in preterm neonates. An LUS higher than 8 was associated with the highest global accuracy (replacement test), suggesting it can be used to guide surfactant administration. Neonatal LUS values of 4 or lower were associated with the highest sensitivity (triage test), suggesting an unlikely need for surfactant in this population.

## Notes

Supplement 1. **eTable 1.** Definitions of the Different Types of Respiratory Failure Used in the Study **eTable 2.** Breakdown of Basic Population Details per Each Recruiting Center **eTable 3.** Main Diagnostic Accuracy Parameters for LUS Thresholds Suitable as Triage Test
eReferences

Supplement 2.
Data Sharing Statement
