# Errors in Figure 1

**DOI:** 10.1001/jamanetworkopen.2024.46284

**Published:** 2024-10-29

In the Original Investigation titled “Quantitative Lung Ultrasonography to Guide Surfactant Therapy in Neonates Born Late Preterm and Later,”^1^ published May 28, 2024, the numbers in Figure 1 were removed from the top lines of the boxes for negative and positive index text results and for received and did not receive surfactant. This article has been corrected online.^1^
