# Supplementary material for: Quantitative Lung Ultrasonography to Guide Surfactant Therapy in Neonates Born Late Preterm and Later
Source: JAMA Netw Open. 2024 May 28;7(5):e2413446. doi: 10.1001/jamanetworkopen.2024.13446 (PMC11134216; doi:10.1001/jamanetworkopen.2024.13446)
Supplement: Supplement 1. — eTable 1. Definitions of the Different Types of Respiratory Failure Used in the Study eTable 2. Breakdown of Basic Population Details per Each Recruiting Center eTable 3. Main Diagnostic Accuracy Parameters for LUS Thresholds Suitable as Triage Test eReferences [file jamanetwopen-e2413446-s001.pdf]

Supplementary Online Content

De Luca D, Bonadies L, Alonso-Ojembarrena A, et al. Quantitative lung ultrasonography to guide surfactant therapy in late preterm and term neonates. *JAMA Netw Open*. 2024;7(5):e2413446. doi:10.1001/jamanetworkopen.2024.13446

eTable 1. Definitions of the Different Types of Respiratory Failure Used in the Study
eTable 2. Breakdown of Basic Population Details per Each Recruiting Center
eTable 3. Main Diagnostic Accuracy Parameters for LUS Thresholds Suitable as Triage Test
eReferences

This supplementary material has been provided by the authors to give readers additional information about their work.

© 2024 De Luca D et al. *JAMA Network Open*.

eTable 1. Definitions of the different types of respiratory failure used in the study. These definitions are those originally issued by the Montreux international consensus.[1]

Lung imaging findings were considered typical according to classical descriptions or international guidelines for their interpretations.[2,3,4] *OI can be calculated by use of 1) arterial or, if arterial values are unavailable, 2) transcutaneous oxygen tension values (with appropriately calibrated transcutaneous devices) or, if these latter are unavailable, 3) oxygen tension values taken from adequately arterialized capillary blood samples.[1] *OI thresholds are the same of those used in pediatric ARDS definition issued from the Pediatric Acute Lung Injury Consensus Conference (PALICC).[5] Common triggers for NARDS in the enrolled population (i.e.: with respiratory failure in the first 72h of life) are those reported by the international NARDS network:[6] sepsis, aspiration (blood, milk or meconium), pneumonia (infectious or biliary), lung hemorrhage, chorioamnionitis, perinatal asphyxia. Definitions of RDS, TTN or NARDS are mutually exclusive.[1]

**RDS**	Clinical signs of respiratory distress appearing within the first 24h of life, with typical lung imaging# as well as complete, sustained, and prompt response to lung recruitment or surfactant (if needed) or both. As additional criterion lamellar body count (if assayed) ≤30 000/mm³. Criteria for NARDS not fulfilled.
**TTN**	Clinical signs of respiratory distress of mild severity (Silverman score ≤3), with typical lung imaging# appearing within the first 24h and resolving within the first 72h of life, needing treatment only with supplemental oxygen or nasal continuous positive airway pressure or both. As additional criterion lamellar body count (if assayed) >30 000/mm³. Criteria for NARDS not fulfilled.
**NARDS**	Fulfilment of all the following criteria, that is:<br>• Acute onset from a known or suspected clinical insult able to trigger NARDS (see legend above)<br>• Absence of any congenital lung anomalies, RDS and TTN (as defined above) as primary cause of the respiratory failure<br>• Diffuse, bilateral and irregular opacities or infiltrates, or complete opacification of the lungs (at chest X-rays or lung ultrasound, whichever is used in clinical routine) not typical of or not fully explained by RDS, TTN, congenital anomalies or local atelectasis.<br>• Absence of any echocardiographic sign of congenital heart disease explaining the lung oedema.<br>• Serious oxygenation impairment defined according to classical oxygenation index (OI)* thresholds (i.e.: Mild NARDS: 4≤OI<8; Moderate NARDS: 8≤OI<16; Severe NARDS: OI≥16)

© 2024 De Luca D et al. *JAMA Network Open*.

eTable 2. Breakdown of basic population details per each recruiting centre.

Data are expressed as number (%), mean (standard deviation) or median [25th – 75th percentile]. Prenatal steroids are considered as two 12mg-betamethasone doses given at least 24h before birth. Postnatal age is considered as the hours of life at the ultrasound examination. Apgar and LUS are dimensionless variables. Centers are anonymized. **Abbreviations**: LUS: lung ultrasound score.

	**A** (n=17)	**B** (n=20)	**C** (n=25)	**D** (n=36)	**E** (n=59)
**Gestational age** Mean (SD), weeks	35.3 (1.7)	34 (1)	37.6 (2.3)	37 (2)	34.8 (2.2)
**Birth weight** Mean (SD), weeks	2470 (485)	2290 (411)	3033 (569)	2865 (580)	2420 (846)
**Male sex** No. (%)	13 (76%)	11 (55%)	12 (48%)	23 (64%)	37 (63%)
**Prenatal steroids** No. (%)	10 (58.9%)	17 (85%)	4 (16%)	12 (33.3%)	17 (28.8%)
**Cesarean section** No. (%)	12 (71%)	15 (75%)	9 (36%)	26 (72%)	34 (58%)
**5' Apgar score** Median [25th – 75th percentile]	9 [8-9]	8 [7-8]	8 [7-9]	8 [7-9]	9 [7-10]
**LUS** Median [25th – 75th percentile]	8 [6-10]	7 [2-9]	9 [5-12]	5 [3-8]	7 [4-9]

© 2024 De Luca D et al. *JAMA Network Open*.

eTable 3. Main diagnostic accuracy parameters for LUS thresholds suitable as triage test (i.e. associated with highest sensitivity). **Abbreviations:** CI: confidence interval; LUS: lung ultrasound score; PV: predictive value.

LUS cut-off	Sensitivity (95%CI)	Specificity (95%CI)	+PV (95%CI)	-PV (95%CI)
**>0**	100 (89-100)%	6.5 (2.8-12.2)%	21.5 (21-22.3)%	100%
**>1**	100 (89-100)%	8 (4-14.2)%	22 (21-23)%	100%
**>2**	100 (89-100)%	20 (13-28)%	24 (23-26)%	100%
**>3**	97 (84-99)%	31 (23-40)%	27 (24-29)%	98 (85-99)%
**>4**	97 (84-99)%	41 (32-50)%	30 (26-33)%	98 (88-99)%
**>5**	94 (79-99)%	47 (38-56)%	31 (27-35)%	97 (88-99)%

© 2024 De Luca D et al. *JAMA Network Open*.

eREFERENCES

1. De Luca D, van Kaam AH, Tingay DG, et al. The Montreux definition of neonatal ARDS: biological and clinical background behind the description of a new entity. *Lancet Respir Med.* 2017; 5: 657–66. doi: 10.1016/S2213-2600(17)30214-X.
2. De Luca D, Baroni S, Vento G, et al. Secretory phospholipase A2 and neonatal respiratory distress: pilot study on broncho-alveolar lavage. *Intensive Care Med.* 2008;34(10):1858-64. doi: 10.1007/s00134-008-1224-3.
3. Gizzi C, Klifa R, Pattumelli MG, et al. Continuous Positive Airway Pressure and the Burden of Care for Transient Tachypnea of the Neonate: Retrospective Cohort Study. *Am J Perinatol.* 2015;32(10):939-43. doi: 10.1055/s-0034-1543988.
4. Singh Y, Tissot C, Fraga MV, et al. International evidence-based guidelines on Point of Care Ultrasound (POCUS) for critically ill neonates and children issued by the POCUS Working Group of the European Society of Paediatric and Neonatal Intensive Care (ESPNIC). *Crit Care.* 2020;24(1):65. doi: 10.1186/s13054-020-2787-9.
5. Khemani RG, Smith LS, Zimmerman JJ, Erickson S, for the Pediatric Acute Lung Injury Consensus Conference Group. Pediatric acute respiratory distress syndrome: definition, incidence, and epidemiology: proceedings from the Pediatric Acute Lung Injury Consensus Conference. *Pediatr Crit Care Med.* 2015; 16 (5 suppl 1): S23–40.
6. De Luca D, Tingay DG, van Kaam AH, et al; Neonatal ARDS Project Collaboration Group. Epidemiology of Neonatal Acute Respiratory Distress Syndrome: Prospective, Multicenter, International Cohort Study. *Pediatr Crit Care Med.* 2022;23(7):524-534.

© 2024 De Luca D et al. *JAMA Network Open*.
