# Supplementary material for: Quantitative Lung Ultrasonography to Guide Surfactant Therapy in Neonates Born Late Preterm and Later
Source: JAMA Netw Open. 2024 May 28;7(5):e2413446. doi: 10.1001/jamanetworkopen.2024.13446 (PMC11134216; doi:10.1001/jamanetworkopen.2024.13446)
Supplement: Supplement 2. — Data Sharing Statement [file jamanetwopen-e2413446-s002.pdf]

Data Sharing Statement

De Luca. Quantitative Lung Ultrasonography to Guide Surfactant Therapy in Late Preterm and Term Neonates. *JAMA Netw Open*. Published May 28, 2024.
doi:10.1001/jamanetworkopen.2024.13446

Data
Data available: Yes
Data types: Deidentified participant data
How to access data: upon reasonable request to the corresponding author (dm.deluca@icloud.com)
When available: With publication

Supporting Documents
Document types: None

Additional Information
Who can access the data: protocol and statistical plan are published and already available online
Types of analyses: for reasonable research projects
Mechanisms of data availability: without investigator support
Any additional restrictions: NA
